# Regional distribution of polymorphisms associated to the disease-causing gene of spinocerebellar ataxia type 3

**DOI:** 10.1007/s00415-024-12829-9

**Published:** 2024-12-12

**Authors:** Tim Lukas Elter, Daniel Sturm, Magda M. Santana, Tamara Schaprian, Mafalda Raposo, Ana Rosa Vieira Melo, Manuela Lima, Berkan Koyak, Demet Oender, Marcus Grobe-Einsler, Sara Lopes, Patrick Silva, Luís Pereira de Almeida, Paola Giunti, Hector Garcia-Moreno, Suran Nethisinhe, Jeroen de Vries, Bart P. van de Warrenburg, Judith van Gaalen, Matthis Synofzik, Ludger Schöls, Kathrin Reetz, Friedrich Erdlenbruch, Heike Jacobi, Jon Infante, Olaf Riess, Thomas Klockgether, ESMI study group, Dagmar Timmann, Andreas Thieme, Jennifer Faber, Jeannette Hübener-Schmid

**Affiliations:** 1 German Center for Neurodegenerative Diseases, Clinical Research, Venusberg-Campus 1/99, 53127 Bonn, Germany; 2 Institute for Medical Genetics and Applied Genomics, University of Tuebingen, Tuebingen, Germany; 3 Center for Rare Disease, University of Tübingen, Tübingen, Germany; 4 Center for Neuroscience and Cell Biology (CNC), University of Coimbra, Coimbra, Portugal; 5 IBMC – Instituto de Biologia Molecular e Celular, i3S – Instituto de Investigação e Inovação em Saúde, Universidade do Porto, Porto, Portugal; 6 Faculdade de Ciências e Tecnologia, Universidade dos Açores, Ponta Delgada, Portugal; 7 Center for Neurology, Department of Parkinson’s Disease, Sleep and Movement Disorders, University Hospital Bonn, University of Bonn, Bonn, Germany; 8 Center for Innovative Biomedicine and Biotechnology (CIBB), University of Coimbra, Coimbra, Portugal; 9 Institute for Interdisciplinary Research, University of Coimbra (IIIUC), Coimbra, Portugal; 10 Faculty of Pharmacy, University of Coimbra (FFUC), Coimbra, Portugal; 11 ViraVector - Viral Vectors for Gene Transfer Core Facility, University of Coimbra, Coimbra, Portugal; 12 Ataxia Centre, Department of Clinical and Movement Neurosciences, UCL Queen Square Institute of Neurology, University College London, London, UK; 13 Department of Neurogenetics, National Hospital for Neurology and Neurosurgery, University College London Hospitals NHS Foundation Trust, London, UK; 14 University Medical Center Groningen, Neurology, Groningen, The Netherlands; 15 Department of Neurology, Donders Institute for Brain, Cognition, and Behaviour, Radboud University Medical Center, Nijmegen, The Netherlands; 16 Department of Neurology, Rijnstate Hospital, Arnhem, The Netherlands; 17 Division Translational Genomics of Neurodegenerative Diseases, Hertie Institute for Clinical Brain Research and Center of Neurology, University of Tübingen, Tübingen, Germany; 18 German Center for Neurodegenerative Diseases (DZNE), Tübingen, Germany; 19 Department of Neurology, RWTH Aachen University, Aachen, Germany; 20 JARA-BRAIN Institute Molecular Neuroscience and Neuroimaging, Research Centre Juelich GmbH and RWTH Aachen University, Aachen, Germany; 21 Department of Neurology and Center for Translational Neuro- and Behavioral Sciences, University Hospital Essen, University of Duisburg, Essen, Germany; 22 Department of Neurology, University Hospital of Heidelberg, Heidelberg, Germany; 23 University Hospital Marqués de Valdecilla-IDIVAL, Santander, Spain; 24 Centro de Investigación Biomédica en Red de Enfermedades Neurodegenerativas (CIBERNED), Universidad de Cantabria, Santander, Spain; 25 Department of Neuroradiology, University Hospital Bonn, Bonn, Germany

**Keywords:** SCA3, Polymorphism, *ATXN3*, Spinocerebellar ataxia, SNP, ASO

## Abstract

**Introduction:**

Knowledge about the distribution and frequency of the respective haplotypes on the wildtype and mutant allele is highly relevant in the context of future gene therapy clinical studies in Spinocerebellar Ataxia Type 3, the most common autosomal dominantly inherited ataxia. Single nucleotide polymorphisms associated to the disease-causing gene, *ATXN3*, have been determined. We wanted to investigate the frequency and regional distribution of two intragenic single nucleotide polymorphisms (SNPs) in a large European SCA3 cohort and their relation to the clinical phenotype.

**Methods:**

The genotypes of the two polymorphisms at base pair positions 987 and 1118 of the *ATXN3* were determined for their co-localization on the normal and expanded allele, respectively, in 286 SCA3 mutation carriers and 117 healthy controls from 11 European sites.

**Results:**

The distribution of genotypes on the expanded allele differed from those of the wildtype allele of SCA3 mutation carriers and of healthy controls, and was mainly influenced by the regional origin. In our cohort, no particular clinical phenotype was associated with any specific haplotype.

**Conclusions:**

Our results confirm distinct allocations of SNPs associated to the expanded *ATXN3*, and accordingly the consideration of allele-specific therapies.

**Supplementary Information:**

The online version contains supplementary material available at 10.1007/s00415-024-12829-9.

## Introduction

Spinocerebellar ataxia type 3, SCA3, also known as Machado-Joseph Disease, MJD, is caused by a coding elongated CAG repeat expansion and is one of the most common autosomal dominantly inherited ataxias [1]. SCA3 has a clinical onset in adulthood and is characterized by progressive ataxia, which can be accompanied by additional neurological symptoms such as oculomotor dysfunction or spasticity [2]. The highest prevalence is found in the Azores (Portugal) [1, 3].

The disease-determining CAG repeat expansion is located on chromosome 14q32.12, in the protein coding region of the *ATXN3* gene [4–6]. Wildtype alleles usually have a length of up to 44 CAG repeats [1, 7, 8]. Due to the CAG repetition on the expanded allele, with around 60–87 repeats, the resulting ATXN3 protein exhibits an expanded polyglutamine (polyQ) tract causing dysfunction of the altered protein [1, 9]. Such mutant ATXN3 is prone to aggregation and responsible for a “toxic gain of function” disturbing cellular homeostasis [10, 11]. Currently, there is no disease-modifying treatment available and the clinical management remains symptomatic and supportive. However, first clinical trials with gene therapy approaches such as antisense oligonucleotides (ASO) have been initiated (https://clinicaltrials.gov, NCT05160558, NCT05822908). ASOs and other approaches including small interfering RNAs (siRNA) aiming at silencing the *ATXN*3 gene are currently promising therapeutic options in SCA3 [12–17]. Importantly, a general aim of gene therapies is to reduce the disease protein in an allele-specific manner to preserve the physiological function of the wildtype allele. Consequently, single nucleotide polymorphisms (SNPs) that are linked to the *ATXN3* gene, especially to the expanded allele, are of particular interest for an allele-specific gene therapy approach. The knowledge about the frequency and regional distribution of the related haplotypes is of central relevance regarding personalized therapies.

Several SNPs associated to the *ATXN3* gene have been described particularly with regard to the expanded, disease-determining allele [18–21]. Two well-described SNPs showed an association with the disease determining allele: first, rs12895357 at base-pair (bp) 916 (c.916G > C; p.Gly306Arg) is historically known and thus also in the present work labeled as bp987, and second, rs7158733 at bp1118 (c.1118C > A; p.Tyr349*) [4, 21–23]. These two SNPs are part of the haplotype described in the two major ancestral origins of SCA3: (i) the Machado lineage, determined by the GTG**GC**A haplotype, which is geographically more restricted to the Azorean island of São Miguel and mainland Portugal, and (ii) the TTA**CA**C haplotype, also known as the Joseph lineage, is found in the ancient mutation origin in Asia, which later spread throughout Europe, especially on the Portuguese mainland and on the Azorean island of Flores [21, 24]. Several studies showed the segregation of the C-A haplotype on the expanded allele in the majority of the families of various ethnic backgrounds including Portugal (Azores and mainland), Brazil, Spain, Taiwan, Germany, Japan, France, UK, India, US, Cambodia, and China [4, 21, 22, 25].

The determination of SNPs is not part of standard diagnostic genetic testing and characterizations of SNPs in large cohorts of SCA3 mutation carriers are up to now missing. In particular, the frequency and regional distribution of the two SNPs linked to the expanded allele across Europe remains elusive, even though allele-specific protein-lowering therapies covering bp987 and bp1118 are already under development [12, 26]. The aim of this work was to study the frequency and regional distribution of the two above-mentioned SNPs (bp987 and bp1118) in a large European cohort of 280 SCA3 mutation carriers and to determine the relation of the intragenic SNPs to the clinical phenotype.

## Methods

### Participants

All participants were included in the longitudinal observational study European Spinocerebellar Ataxia Type 3/Machado-Joseph-Disease Initiative (ESMI) and gave their written informed consent according to the declaration of Helsinki. The inclusion criteria were as follows: (i) age ≥ 18 years, (ii) known SCA3 mutation carrier, or first-degree relative of a SCA3 mutation carrier or healthy control. Participants were recruited at 11 European research centers (London, UK; Bonn, Aachen, Essen, Tübingen and Heidelberg, Germany; Coimbra and the Azores, Portugal; Nijmegen and Groningen, The Netherlands; Santander, Spain). EDTA blood samples were taken following a standardized protocol [27]. In total, DNA samples from 286 SCA3 mutation carriers and 117 healthy controls were genotyped for the SNPs rs12895357 at bp987 and rs7158733 at bp1118 of the *ATXN3* gene. As demographic and genetic data, age at baseline and sex as well as the CAG repeat length of both alleles were extracted. Determination of CAG repeat length was assessed centrally for all participants at the Institute of Medical Genetics and Applied Genomics, Tübingen.

Two SCA3 mutation carriers were homozygous for the disease-specific mutation. They were excluded from the main analysis to maintain statistical conformity. Their descriptive information is provided in a separate paragraph of the results’ section.

Clinical assessment included the Scale for the Assessment and Rating of Ataxia (SARA) [28] and the Inventory of Non-Ataxia Signs (INAS) [29]. The INAS assesses the presence *vs*. absence of the following neurological symptoms: hyperreflexia, areflexia, extensor plantar reflex, spasticity, paresis, muscle atrophy, fasciculations, myoclonus, rigidity, chorea/dyskinesia, dystonia, resting tremor, sensory symptoms, urinary dysfunction, cognitive dysfunction and brainstem oculomotor signs, the latter comprising ophthalmoparesis on horizontal and/or vertical gaze and/or slowing of saccades. The INAS count sums up the number of present additional neurological symptoms, ranging from 0 (no other neurological symptom) to a maximum of 16 neurological symptoms.

Age of ataxia onset was defined as the reported first occurrence of gait disturbances. For SCA3 mutation carriers, not yet experiencing gait disturbances or with missing information about the age of onset (*n* = 16), the age of onset was calculated on the basis of CAG repeat and age [30].

Mutation carriers with a SARA sum score of < 3 at baseline were categorized as pre-ataxic, according to the established SARA sum score cut-off, irrespective of SARA scores at follow-up visits. To assess the clinical phenotype, we examined the SCA3 mutation carrier within their haplotype at bp987 and bp1118.

### Genetic analysis

The CAG repeat of *ATXN3* was amplified using CAG repeat flanking primers [12]. Shortly, 150 ng of genomic DNA were amplified by a standard polymerase chain reaction (PCR) followed by capillary electrophoresis using the Beckman Coulter Fragment Analysis Software (Beckman Coulter).

SNPs at bp987 and bp1118 were determined in an allele-specific manner using fragment analyses. Therefore, PCR reactions were carried out using two different reverse primers that differ at the respective base position of the SNP bp 987 or bp1118, each labeled with a different fluorophore (forward primer 5′-CCAGTGACTACTTTGATTCG-3′ for both bp987 and bp1118 SNPs; reverse primer bp987 wildtype 5′-IRD700-ACTCTGTCCTGATAGGTCCC**C**−3′; reverse primer bp987 SNP 5′-Cy5-ACTCTGTCCTGATAGGTCCC**G**−3′; reverse primer bp1118 wildtype 5′-IRD700-GCAAAAATCACATGGAGCTC**G**−3′; reverse primer bp1118 SNP 5′-Cy5-GCAAAAATCACATGGAGCTC**T**−3′). 150 ng of genomic DNA was amplified in a standard PCR reaction containing 5% HiDi™ formamide (bp987; Life Technologies LTD, Warrington, UK) or 4 µl 5 × Q-Solution (bp1118; Qiagen, Hilden, Germany), respectively, and 10 µM of each primer. The optimal annealing temperatures were set at 59.5 °C for bp987 and 58 °C for bp1118, respectively. PCR products were separated by size using capillary electrophoresis determining the respective fluorophores using Beckman Coulter Fragment Analysis Software (Beckman Coulter, Brea, US).

### Statistical analysis

Statistical analysis was performed with R (version 4.2.1), except for the pie-charts that were created with SPSS (Version 23.0.0.2). The annual SARA progression was calculated as the average annual rate between SARA at baseline and the last visit. Chi-square test was used to determine Hardy–Weinberg equilibrium (HWE).

We applied the exact Fisher test to study the distributions of SNPs at bp987 and bp1118 between the wildtype and the expanded alleles of SCA3 mutation carriers and healthy controls and also between ataxic and pre-ataxic SCA3 mutation carriers.

To study group differences between the subgroups of controls and SCA3 mutation carriers, and pre-ataxic and ataxic SCA3 mutation carriers, the exact Fisher test was used for the variable sex, and the Mann–Whitney test for the variables CAG repeat, SARA sum score, SARA-annual progression, INAS count and disease duration at baseline. Group differences of age at baseline and age of onset were determined using a T-test.

The relation between the four different haplotypes of the expanded allele within the group of ataxic SCA3 mutation carriers was tested with the Fisher’s test for sex, One-way ANOVA for age at baseline, reported and age of onset. Kruskal–Wallis Test was used for the variables CAG repeat length, SARA sum score, SARA-annual progression, INAS count and disease duration at baseline. Additionally, closed post-hoc test was done for further analysis of SARA sum score.

Furthermore, an exact Fisher test was used to compare the presence and absence of single INAS items between the four different haplotypes of the expanded allele within the group of ataxic SCA3 mutation carriers. Subsequently, those items that showed a significant difference, were subjected to a closed post-hoc test with a corrected alpha, due to alpha error correction for $p=\alpha /(n-1)$, with *n* equals the number of considered items, to study the relation of haplotypes and the respective INAS items.

## Results

### Cohort characterization

A total of 226 ataxic SCA3 mutation carriers, 54 pre-ataxic SCA3 mutation carriers and 113 healthy controls were included in the final analysis. Table 1 gives an overview of the demographic, genetic and clinical data of controls and all SCA3 mutation carriers. Eight subjects (4 SCA3 mutation carriers and 4 healthy controls) were excluded from the final analysis due to missing clinical data.

**Table 1:** Clinical and demographic characteristics of SCA3 mutation carriers and healthy controls

	Controls	SCA3	Sign	*p* value
*N*	113	280		n.a
*N* male/female (%)	50/63 (44.2/55.8)	138/142 (49.3/50.7)		0.38^1^
Age at baseline Mean [SD]	45.5 [14.2]	48.2 [13.0]		0.07^2^
CAG repeats longer allele Median [IQR]	24.0 [23.0, 27.0]	69.0 [66.0, 71.0]	**	< 0.001^3^
Age of onset Mean [SD]	n.a	40.35 [9.78]		n.a
SARA sum score Median [IQR]	0.0 [0.0, 0.50]	9.5 [4.0, 17.0]	**	< 0.001^3^
SARA sum score annual progression Median [IQR]	0.0 [0.0, 0.0]	0.81 [0.0, 2.1]	**	< 0.001^3^
INAS count Median [IQR]	1.0 [0.0, 1.0]	4.5 [3.0, 7.0]	**	< 0.001^3^
Disease duration at baseline, in years^4^ Median [IQR]	n.a	7.9 [3.6, 14.6]		n.a

*n.a.* not applicable, *sign.* Significance, *IQR* interquartile range, *SD* standard deviation

*p < 0.05; **p < 0.001

^1^Fisher Test

^2^t-Test

^3^Mann-Whitney Test

^4^Disease duration is defined as the time from the age of onset (detailed description is given in the Methods section) until baseline visit, in years

The two patients who were homozygous for the disease-causing gene with two expanded alleles were excluded from the main analysis. Their demographic, genetic and clinical information is given separately.

Frequencies of both, the whole cohort (including healthy controls and mutations carriers) and control group, were in Hardy–Weinberg equilibrium (SNP 987: all *p* = 0.5998; CNTR *p* = 0.144; SNP 1118: all *p* = 0.158; CNTR *p* = 0.718) Deviation from Hardy–Weinberg equilibrium were found in mutation carriers for all analyzed SNPs (SNP 987 *p* = 0.0086; SNP 1118 *p* = 0.0144).

### Distribution and frequency of SNPs at bp987 and bp1118 on the wildtype and expanded ATXN3 alleles

**Fig. 1:**
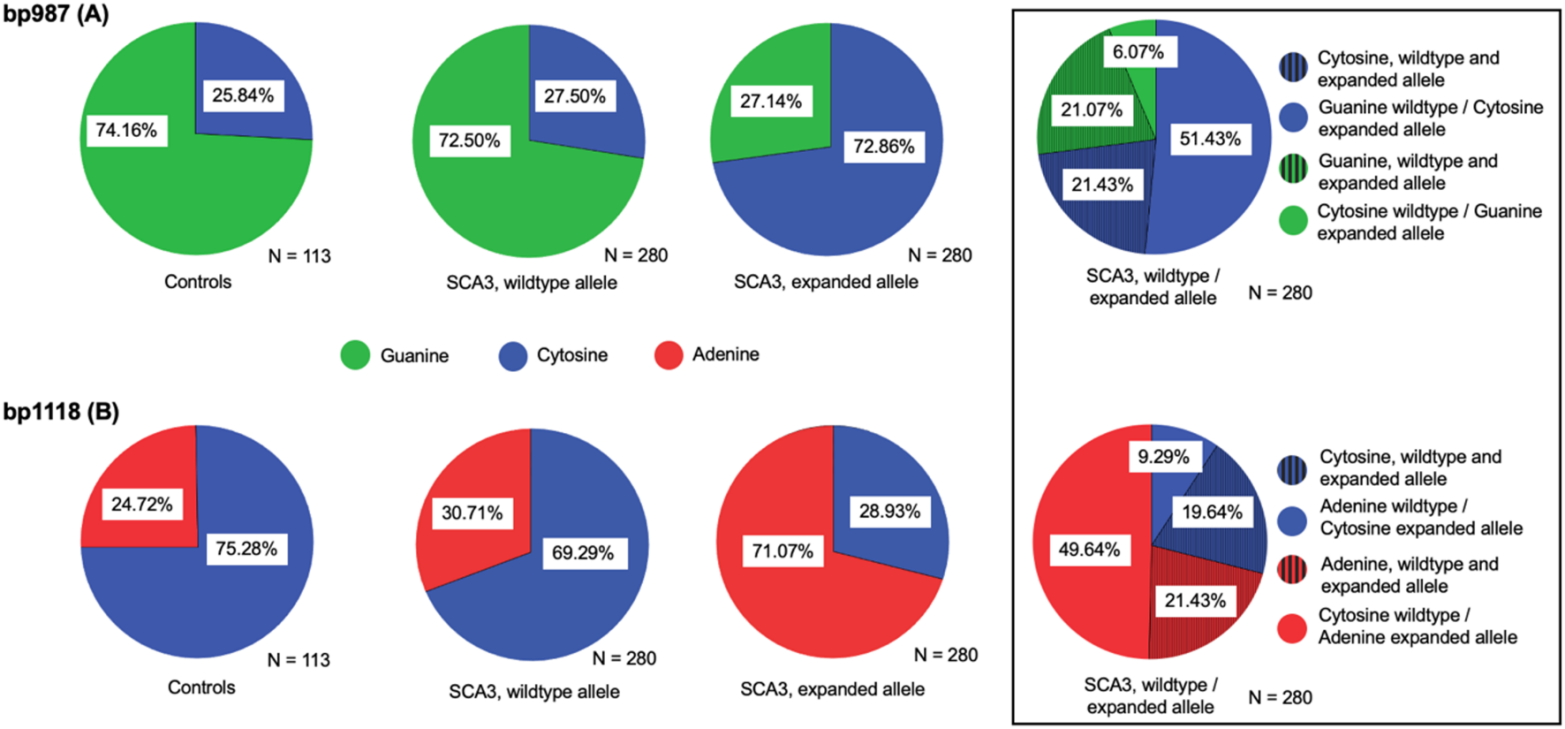
Relative frequencies of the SNP distributions at bp987 and bp1118 on the wildtype and expanded alleles. The relative frequencies of Guanine (green) and Cytosine (blue) at bp987 (**A**) and of Adenine (red) and Cytosine (blue) at bp1118 (**B**) are given for healthy controls as well as the wildtype and expanded allele in SCA3 mutation carriers, respectively. In the box on the far left, the distribution of the extended allele is further categorised: The respective proportion in which the wildtype and the expanded allele do not differ, e.g. do have the same nucleobase, is hatched. The distribution in controls is comparable to those of the wildtype allele in SCA3, while both are significantly different from the distribution on the expanded allele of SCA3 (*p* < 0.001)

The distribution of the wildtype alleles of the SCA3 mutation carriers did not differ statistically from healthy controls. However, the distributions of SNPs in healthy controls and wildtype allele in SCA3 is significantly different from those observed on the expanded allele in SCA3 (*p* < 0.001, Fig. 1, Supplementary Table 1). For the SNP at position bp987 the majority of healthy controls and the majority of wildtype alleles in SCA3 showed Guanine (HC: 74.16%; wildtype allele SCA3: 72.5%), compared to only one-fourth of the expanded alleles in SCA3 (27.14%). For the SNP at bp1118 the majority of healthy controls as well as wildtype alleles in SCA3 showed Cytosine (HC: 75.28%, wildtype SCA3: 69.29%), compared to only 28.93% of the expanded alleles in SCA3 (Fig. 1). Genotype distribution in expanded alleles in ataxic and pre-ataxic SCA3 mutation carriers did not differ (Supplementary Table 1).

Moreover, we studied the percentage, where the SCA3 wildtype allele differed from the expanded allele: In 57.5% the wildtype SNP differed from the expanded allele at bp 987, and in 58.9% at bp1118 (Fig. 1, right box).

The two homozygous SCA3 mutation carriers that were excluded from the final analysis had an early age of onset (23 and 30 years) and a high SARA sum score at baseline examination (28.5 and 23) with a disease duration of 16.05 and 17.02 years, respectively. They showed Cytosine—Guanine and Guanine—Guanine at bp987, and both Adenine—Adenine at bp1118. The CAG-repeats on the expanded alleles were 64/64 and 60/66, respectively.

### Distribution of the SNPs at wildtype and expanded allele per research center

**Fig. 2:**
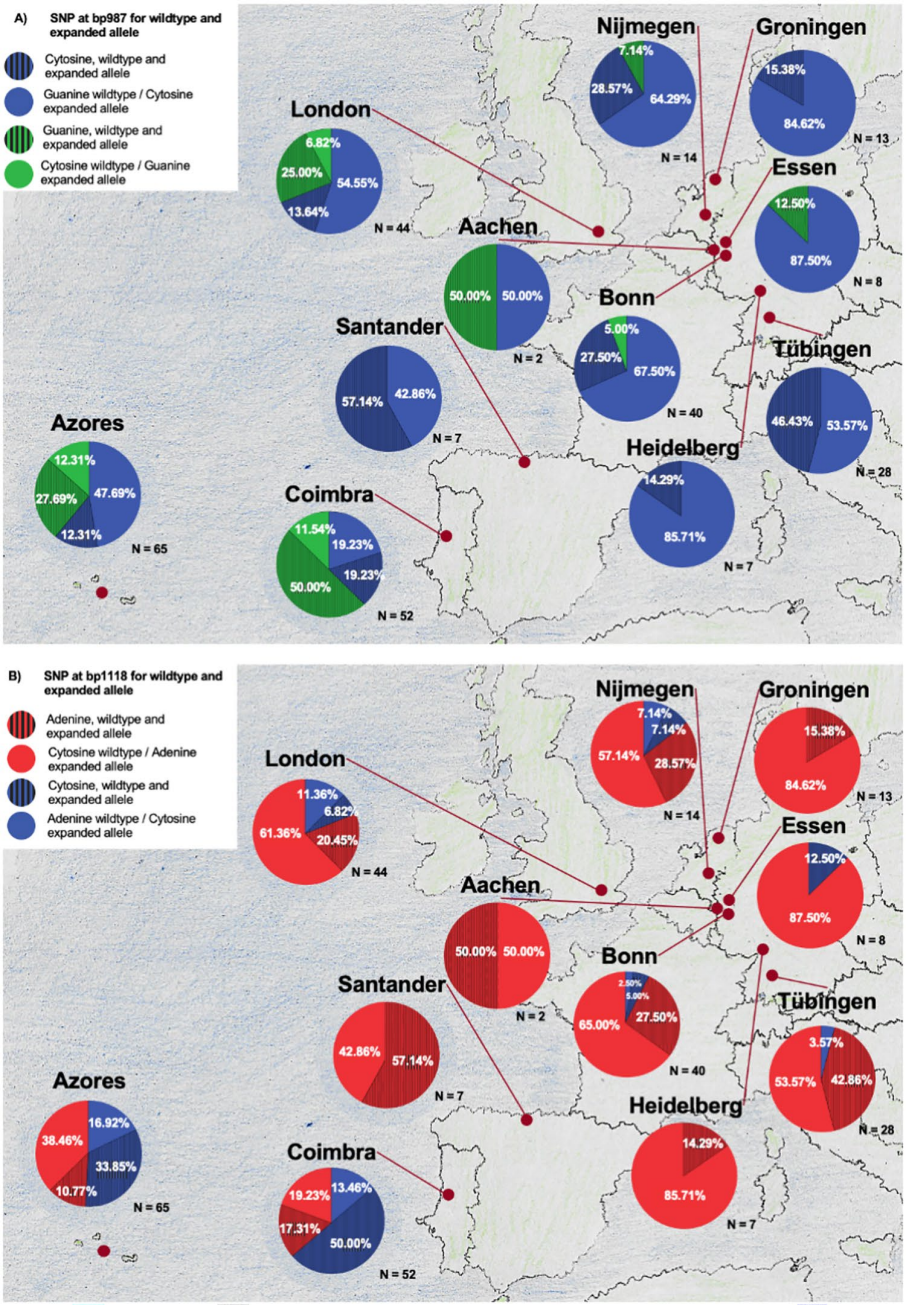
Relative frequencies of the SNPs at bp987 (**A**) and bp1118 (**B**) of the wildtype and expanded allele for each European site. Color codes the nucleobase at the expanded allele: cytosine in blue, guanine in green and adenine in red. Full-colored areas indicate the proportion with a different nucleobase at wildtype and expanded allele, while the hatched areas indicate the presence of the same nucleobase at wildtype and expanded allele

The majority of SCA3 mutation carriers at continental European research centers show on the expanded allele at bp987 Cytosine and Guanine on the wildtype. However, Portuguese research centers in Coimbra and on the Azores as well as London showed a different vice versa pattern. A similar constellation is depicted for the SNP at bp1118 with mostly Adenine on the expanded allele and Cytosine on the wildtype allele. Again, the Portuguese research centers in Coimbra and on the Azores showed a larger proportion of Cytosin on the expanded allele (Fig. 2). At the German site Aachen, only 2 participants were included, thus we do not recognize the proportions here as a trend.

### Distribution of haplotypes and association with demographic, genetic and clinical data

**Fig. 3:**
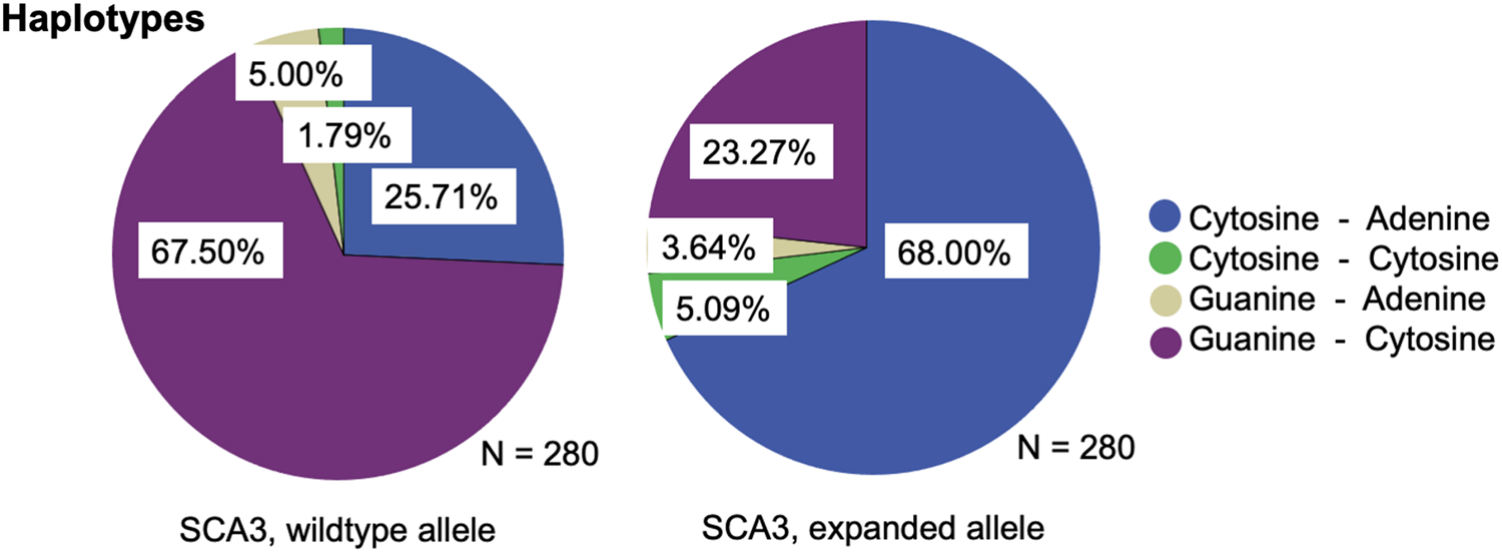
Relative frequencies of the haplotypes of the wildtype and expanded allele at bp987 and bp1118 for SCA3 mutation carriers. Blue areas represent the frequency of Cytosine (bp987)–Adenine (bp1118), green areas the frequency of Cytosine–Cytosine, ochre areas the frequency of Guanine–Adenine and purple areas the frequency of Guanine–Cytosine, respectively

Four different haplotypes, defined by the SNPs at positions bp987 and bp1118, were found in both wildtype and expanded alleles of the analyzed SCA3 mutation carriers. The haplotypes of the wildtype allele in SCA3 mutation carriers present a frequency of 67.5% Guanine–Cytosine, 25.71% Cytosine–Adenine, 5.0% Guanine–Adenine and 1.79% Cytosine–Cytosine at bp987 and bp1118, while expanded alleles in SCA3 mutation carriers at bp987 and bp1118 demonstrate a frequency of 68.0% Cytosine–Adenine, 23.27% Guanine–Cytosine, 5.09% Cytosine–Cytosine and 3.64% Guanine–Adenine (Fig. 3).

Genotypic and clinical evaluation of the cohort showed significant differences between SCA3 mutation carriers and controls in CAG repeat lengths, SARA sum score and annual progression and INAS count (*p* < 0.001, Table 1). Pre-ataxic and ataxic SCA3 mutation carriers showed significant differences regarding age at baseline, SARA sum score and SARA progression, INAS count as well as the disease duration at baseline (*p* < 0.001, supplementary Table 2).

SARA annual progression, INAS count, age of disease onset and disease duration at baseline as well as the CAG repeat length of the longer allele were similar between the four haplotypes in SCA3 (Table 2, supplementary Table 3).

**Table 2:** Clinical and demographic characteristics of SCA3 mutation carriers for each haplotype of the expanded allele (bp987 and bp1118)

	Haplotype, expanded allele bp987–bp1118				Sign	*p* value
	Cytosine–Adenine	Guanine–Cytosine	Cytosine–Cytosine	Guanine–Adenine		
*N*	189	66	15	10		n.a
*N* male/female (%)	96/93 (50.8/49.2)	30/36 (45.5/54.5)	8/7 (53.3/46.7)	4/6 (40/60)		0.80^1^
Age at baseline Mean [SD]	48.14 [12.41]	48.23 [14.24]	45.83 [12.72]	51.87 [16.14]		0.73^2^
CAG repeats longer allele Median [IQR]	69.00 [66.00, 71.00]	70.00 [66.00, 71.75]	70.00 [67.50, 72.00]	67.50 [66.25, 70.00]		0.61^3^
Age of onset Mean [SD]	40.75 [9.36]	40.64 [10.84]	36.67 [6.77]	36.20 [13.16]		0.23^2^
SARA sum score Median [IQR]	9.50 [4.00, 16.00]	9.00 [4.00, 17.25]	13.50 [8.00, 23.75]	20.00 [15.62, 22.25]	*	0.01^3^
SARA sum score annual progression Median [IQR]	0.86 [0.00, 1.98]	0.56 [− 0.38, 2.19]	2.76 [0.52, 4.07]	0.66 [0.28, 1.67]		0.21^3^
INAS count Median [IQR]	4.00 [2.00, 7.00]	5.00 [3.00, 6.75]	4.00 [3.50, 5.50]	8.00 [5.50, 9.00]		0.09^3^
Disease duration at baseline, in years^4^ Median [IQR]	8.03 [3.40, 13.84]	8.36 [2.98, 13.52]	10.87 [5.37, 18.77]	17.50 [12.05, 21.99]		0.59^3^

*n.a.* non applicable, *sign.* Significance, *IQR* interquatile range, *SD* standard deviation; *p < 0.05

^1^Fisher-Test

^2^One-way Anova

^3^Kruskal–Wallis Test

^4^Disease duration is defined as the time from the age of onset (detailed description is given in the Methods section) until baseline visit, in years

SARA sum score in the group of all SCA3 mutation carriers (Table 2) and the subgroup of ataxic SCA3 mutation carriers (supplementary Table 3) showed a statistically significant difference between the haplotypes (*p* < 0.05; Table 2; supplementary Table 3). The post hoc testing revealed a statistically higher mean SARA sum score in ataxic SCA3 carriers with the haplotype Guanine—Adenine (mean SARA = 20.00, *N* = 10) and the haplotype Cytosine—Adenine (mean SARA 11.00, *N* = 150) (Supplementary Fig. 1 and Supplementary Table 3).

**Fig. 4:**
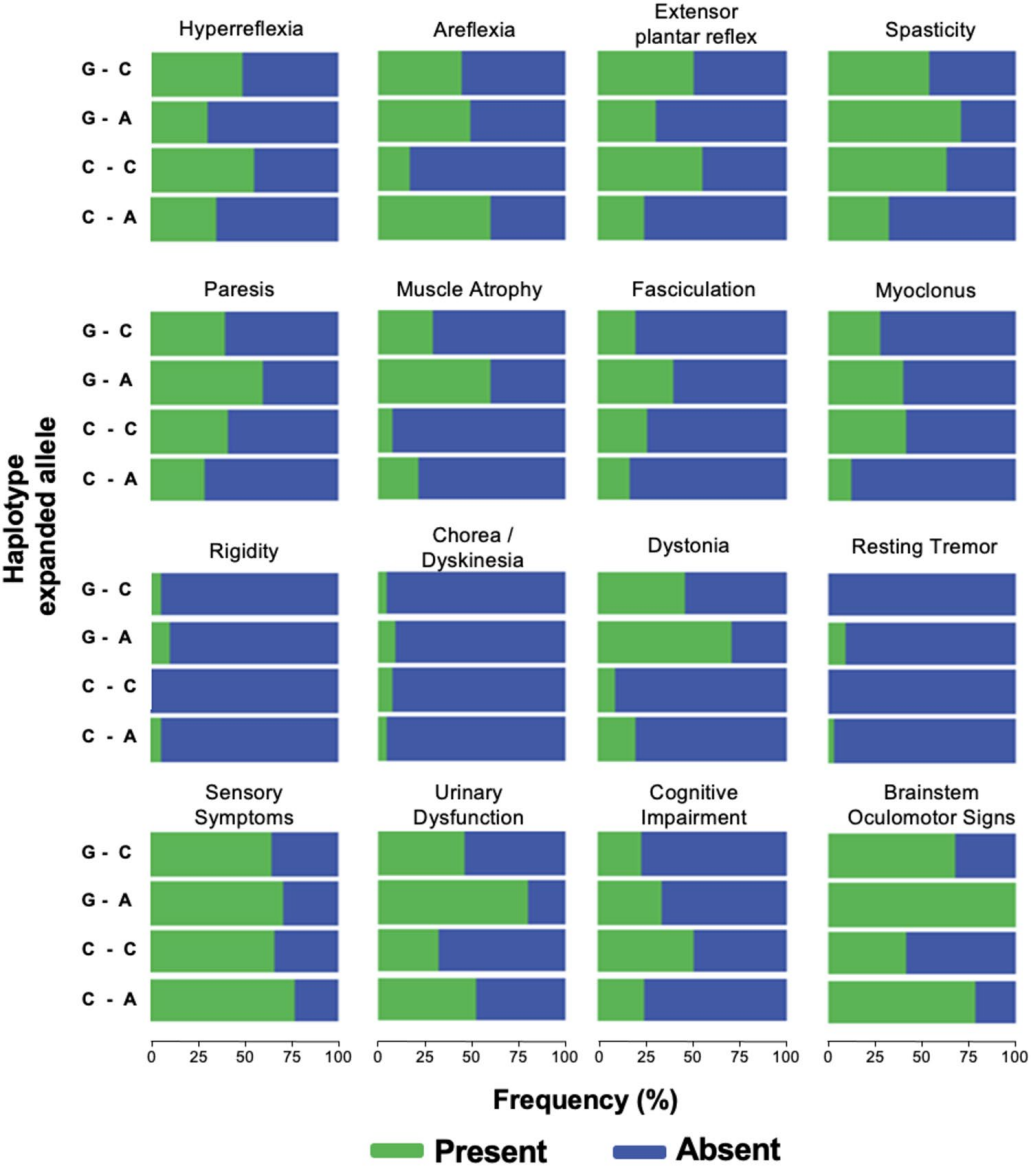
Relative frequencies of neurological, non-ataxia signs. The relative frequencies of additional neurological signs other than ataxia are given for the four haplotypes of the expanded allele (bp987 and bp1118) respectively: Guanine–Cytosine (G–C, N = 54), Guanine–Adenine (G–A, *N* = 10), Cytosine–Cytosine (C–C, *N* = 12) and Cytosine–Adenine (C–A, *N* = 150). Bars indicating the presence of each item are colored in green, and the remaining proportion with absence of the respective sign is marked in blue

To further study potentially different phenotypes related to the analyzed SNPs we compared the distribution of INAS subitems among the haplotypes of the expanded allele in the group of ataxic SCA3 mutation carriers (Fig. 4; supplementary Table 4). Areflexia (C–A *versus* C–C), extensor plantar reflex (C–A *versus* G–C), spasticity (C–A *versus* G–C), muscle atrophy (C–A *versus* G–A), myoclonus, dystonia (C–A *versus* G–C, C–A *versus* G–A, C–C *versus* G–A) and brainstem oculomotor signs (C–A *versus* C–C; C–C *versus* G–A) showed a significant difference between the four haplotypes of the expanded allele (Supplementary Tables 4 and 5).

### Distribution of the haplotypes of the expanded allele per research center

**Fig. 5:**
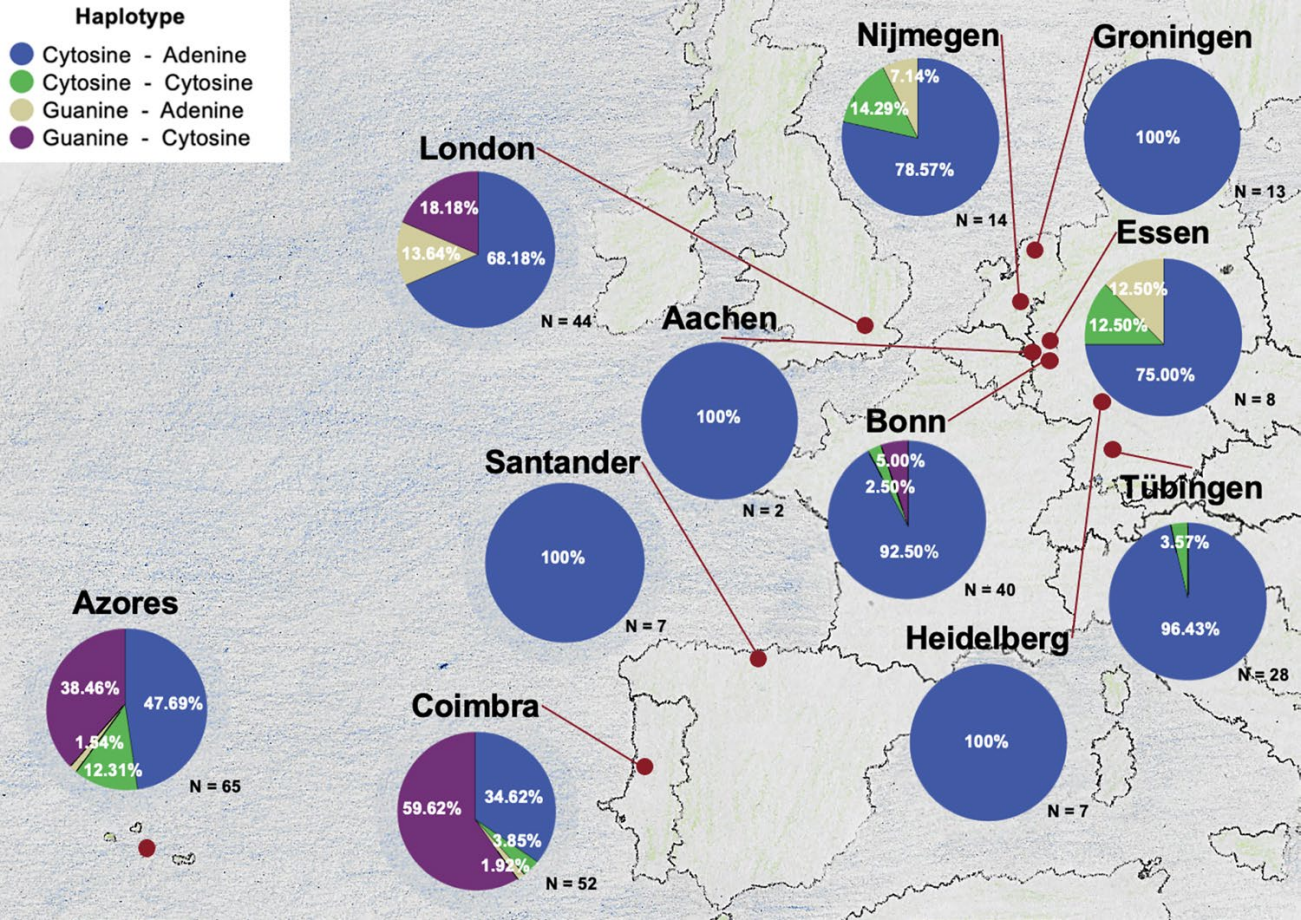
Relative frequencies of haplotypes at SNP bp987–bp1118 on the expanded allele for each European site. The blue area represents the frequency of Cytosine (bp987) –Adenine (bp1118), the green area the frequency of Cytosine–Cytosine, the ochre area the frequency of Guanine–Adenine and the purple area the frequency of Guanine–Cytosine, respectively

To study the regional distribution in Europe, frequencies of haplotypes of the SCA3 expanded allele at bp987 and bp1118 were calculated for each research center separately (Fig. 5). In Portugal, SCA3 mutation carriers from the Azores (*N* = 65) and from Coimbra, mainland Portugal (*N* = 52) showed all possible haplotypes with a particularly high proportion of G–C (Fig. 5, Supplementary Table 6).

## Discussion

To explore the potential of well-known SNPs (bp987, bp1118) in *ATXN3* for allele-specific therapies, such as ASOs, we studied the frequency and regional distribution in a large European cohort of > 200 SCA3 mutation carriers. In line with previous studies, the distribution of haplotypes on the expanded allele showed a considerable difference from those of wildtype alleles of SCA3 mutation carriers and of healthy controls. Additionally, this distribution across Europe was mainly influenced by regional origin. The haplotype of the two intragenic SNPs at bp987 and bp1118 is known to be linked specifically to the CAG expansion in around 70–72% of the mutation carriers [4, 21, 22]. Similar results were found in our study in which 68% of the expanded alleles presented the haplotype C-A at bp987-1118, whereas 67.5% of the wildtype alleles in mutation carriers showed the G-C haplotype. Our comprehensive European haplotyping is in line with previous smaller or less regional distributed descriptions of haplotypes in SCA3 [21]. While the eight German, Dutch and Spanish research centres exhibited limited to no variation with a predominance of the C–A haplotype, associated to the Joseph lineage, of 92.8% to 100%, this haplotype was less frequent in the UK (68%), the Azores (48%) and the Portugal mainland (35%). In contrast, the latter three research centres showed an increased proportion of the Haplotype G–C (18.2%, 38.5% and 59.6%, respectively), associated to the Machado lineage. The haplotypes C–C and G–A were found less frequent with a specific regional origin. In previous studies, the G–A haplotype was described in one family from Morocco, French Guyana and two families from the United States. In addition, the C–C haplotype was not described earlier in SCA3 mutation carriers [21].

Our results provide for the first time a comprehensive overview of the European distribution, where regional differences may partially be explained by different main founder mutations. As highly standardized clinical data were available, we could for the first time analyse the relation between haplotypes and the clinical phenotype. Our data demonstrated, that the SARA sum score and the distribution of INAS subitems were slightly significantly differently distributed among the different haplotypes. Generally, patients with the most common haplotype C–A at the expanded allele demonstrated less frequently pathological extensor plantar reflexes and spasticity compared to the second most often haplotype G–C. Importantly, SCA3 mutation carriers carrying haplotypes C–A and G–C had similar numbers of CAG repeats in the expanded allele, providing evidence that this effect is not biased by different repeat length. Even though, differences in haplotype expression are conceivably related to different phenotypes and symptoms due to ATXN3 isoforms caused by SNPs. It is well known that the SNP at bp1118 leads to a premature stop codon and therefore, to a shorter ATXN3 isoform, known as ATXN3a short (ATXN3aS) [31]. The two most expressed ATXN3 isoforms in the brain ATXN3a short (ATXN3aS, harbouring the A at bp1118) and ATXN3a long (ATXN3aL, harbouring the wildtype sequence with C at bp1118) [32], demonstrated that the isoform ATXN3aS showed shorter half-life, faster degradation by the autophagic as well as proteasomal pathways and formation of larger aggregates compared to ATXN3aL [31]. However, in summary, we could not identify reliable overarching clusters of phenotypic features related to one haplotype. Nevertheless, the haplotypes C–C and G–A were underrepresented, thus further studies with an increased number of SCA3 mutation carriers, in particular of those haplotypes, are needed.

Surprisingly, genotype analyses revealed that only around 60% of SCA3 mutation carriers will benefit from an allele-specific protein-lowering therapy as 40% harbours the same nucleobase at the wildtype and the expanded allele. ASOs and siRNAs aiming at silencing the *ATXN3* gene are currently promising therapeutic options in SCA3 [12–17]. Because of autosomal-dominantly inheritance, most SCA3 mutation carrier represent only one pathogenic expanded allele, therefore an allele-specific gene therapy approach is of particular interest to preserve the physiological function of the wildtype allele [31, 33]. Allele-specificity can be achieved by using SNPs that are specifically linked to the expanded allele. As ASOs and siRNA are short (12–25 nucleotides long) in size, only one or two very closely located SNPs can be targeted by this allele-specific strategy [32, 33, 34, 35]. Therefore, a haplotype-based stratification strategy is not helpful to develop new allele-specific therapies. To get more insights into the use of SNP for allele-specific targeting, we especially focused on the genotype of SNP 987 and 1118 in our large European SCA3 cohort. On average our data with approximately 68% are in line with previous reports of 70–72% of Cytosine for bp987 and Adenine for bp1118 [4, 21, 22, 25]. However, the proportion of SCA3 mutation carriers which are non-treatable with an allele-specific therapy considering the SNPs bp987 and bp1118 since their nucleobases do not differ between wild type and expanded allele differs strongly depending mainly on the origin (in our study between 12 and 69%). There will be SCA3 mutation carrier (in our study between 12 and 69%, depending on the regional origin) which are non-treatable with an allele-specific therapy considering SNPs at bp987 and bp1118 since their nucleobases do not differ between wildtype and expanded allele. Therefore, there is an urgent need to further characterize the allele-specificity of other *ATXN3* specific SNPs in large, regional wide-spread SCA3 cohorts. In summary, with allele-specific protein-lowering therapies under investigation, there is an urgent need to understand the worldwide distribution of SNPs linked to the expanded allele in SCA3. Currently, SNP determination within *ATXN3* is not part of the standard diagnostic genetic CAG repeat evaluation. Our study provided important information about the European distribution of the two examined SNPs, bp987 and bp1118, and might therefore be of help by providing a trial-ready cohort for allele-specific, protein-lowering therapies.

## Supplementary Information

Below is the link to the electronic supplementary material.

Supplementary file1 (DOCX 141 KB)

## Data Availability

Data will be made available upon reasonable request and as patient consent allow.

## Abbreviations

SNPs: Single nucleotide polymorphism
SCA3: Spinocerebellar ataxia type 3
polyQ Tract: Polyglutamine tract
ASO: Antisense oligonucleotide
ANOVA: Analysis of variance
C: Cytosine
A: Adenine
G: Guanine
SARA: Scale for the assessment and rating of ataxia
INAS: Inventory of non-ataxia signs
