# Supplementary material for: Regional distribution of polymorphisms associated to the disease-causing gene of spinocerebellar ataxia type 3
Source: J Neurol. 2024 Dec 12;272(1):54. doi: 10.1007/s00415-024-12829-9 (PMC11638412; doi:10.1007/s00415-024-12829-9)
Supplement: Supplementary file 1 — Supplementary file1 (DOCX 141 KB) [file 415_2024_12829_MOESM1_ESM.docx]

**Supplement**

*Supplementary Table 1.* **Distribution of SNPs at bp987 and bp1118 for control wildtype alleles, SCA3 mutation carrier wildtype and expanded alleles**

| **base pair (bp)** | **Group1** | **Group2** | **sign.** | | **p-value^4^** |
| --- | --- | --- | --- | --- | --- |
|  | **Controls** | **SCA3, wildtype** |  | |  |
| **bp987** | G^1^: 74.16 % C^2^: 25.84 % | G: 72.50 % C: 27.50 % |  | | 0.52 |
| **bp1118** | C : 75.28 % A^3^: 24.72 % | C: 69.29 % A: 30.71 % |  | | 0.07 |
|  |  |  |  | |  |
|  | **Controls** | **SCA3, expanded** |  | |  |
| **bp987** | G: 74.16 % C: 25.84 % | G: 27.14 % C: 72.86 % | ** | | <0.001 |
| **bp1118** | C: 75.28 %. A: 24.72 % | C: 28.93 % A: 71.07 % | ** | | <0.001 |
|  |  |  |  | |  |
|  | **SCA3, wildtype** | **SCA3, expanded** |  | |  |
| **bp987** | G: 72.50 % C: 27.50 % | G: 27.14 % C: 72.86 % | ** | | <0.001 |
| **bp1118** | C: 69.29 % A: 30.71 % | C: 28.93 % A: 71.07 % | ** | | <0.001 |
|  | **Ataxic SCA3, wildtype** | **Pre-ataxic SCA3, wildtype** |  | |  |
| **bp987** | G: 71.7 %  C: 28.3 % | G: 75.9 %  C: 24.1 % |  | | 0.61 |
| **bp1118** | C: 68.6 %. A: 31.4 % | C: 72.2 % A: 27.8 % |  | | 0.74 |
|  |  |  |  | |  |
|  | **Ataxic SCA3, expanded** | **Pre-ataxic SCA3,**  **expanded** |  | |  |
| **bp987** | G: 28.3 %  C: 71.7 % | G: 22.2 %  C: 77.8 % |  | | 0.40 |
| **bp1118** | C: 29.2 % A: 70.8 % | C: 27.8 % A: 72.2 % |  | | 1.00 |
| ^1^Guanine; ^2^Cytosine; ^3^Adenine; ^4^ Fisher-test; sign. = significance; ** p < 0.001 | | | |  |  |

*Supplementary Table 2.* **Clinical and demographic characteristics of pre-ataxic and ataxic SCA3 mutation carriers**

|  | **Pre-ataxic SCA3** | **Ataxic SCA3** | **sign.** | **p-value** |
| --- | --- | --- | --- | --- |
| **N** | 54 | 226 |  | n.a. |
| **N male / female (%)** | 21 / 33 (38.9 / 61.1) | 117 / 109 (51.8 / 48.2) |  | 0.10^1^ |
| **Age at baseline**  mean [SD] | 34.10 [8.59] | 51.59 [11.47] | ** | < 0.001^2^ |
| **CAG repeats longer allele**  median [IQR] | 68.50 [66.00, 70.00] | 69.00 [66.00, 71.00] |  | 0.20^3^ |
| **Age of onset**  mean [SD] | 42.38 [8.46] | 39.86 [10.03] |  | 0.35^2^ |
| **SARA sum score**  median [IQR] | 1.00 [0.00, 2.00] | 12.00 [8.00, 19.50] | ** | < 0.001^3^ |
| **SARA sum score**  **annual progression**  median [IQR] | 0.00 [0.00, 0.49] | 1.07 [0.00, 2.38] | ** | < 0.001^3^ |
| **INAS count**  median [IQR] | 1.00 [0.00, 2.00] | 5.00 [4.00, 7.00] | ** | < 0.001^3^ |
| **Disease duration at baseline, in years^4^**  median [IQR] | -8.28 [-12.63, -5.71] | 10.89 [6.09, 17.22] | ** | < 0.001^3^ |

^1^Fisher-Test; ^2^one-way Anova; ^3^Kruskal-Wallis-Test; ^4^disease duration is defined as the time from the age of onset (detailed description is given in the Methods section) until baseline visit, in years; n.a. = not applicable; sign. = significance; IQR = interquartile range; SD = standard deviation; ** p < 0.001

*Supplementary Table 3.* **Clinical and demographic characteristics of ataxic SCA3 mutation carriers, divided per haplotype of the expanded allele**

|  | **Haplotype, expanded allele bp987 – bp1118** | | | |  |  |
| --- | --- | --- | --- | --- | --- | --- |
|  | Cytosine - Adenine | Guanine - Cytosine | Cytosine - Cytosine | Guanine - Adenine | sign. | p-value |
| **N** | 150 | 54 | 12 | 10 |  | n.a. |
| **N male / female (%)** | 81 / 69 (54 / 46) | 25 / 29 (46.6 / 53.7) | 7 / 5 (58.3 / 41.7) | 4 / 6 (40 / 60) |  | 0.66^1^ |
| **Age at baseline**  mean [SD] | 51.78 [10.52] | 51.32 [13.48] | 50.23 [10.00] | 51.87 [16.14] |  | 0.97^2^ |
| **CAG repeats longer allele**  median [IQR] | 69.00 [66.00, 71.00] | 70.00 [65.25, 72.00] | 71.00 [68.50, 72.00] | 67.50 [66.25, 70.00] |  | 0.47^3^ |
| **Age of onset**  mean [SD] | 40.32 [9.38] | 40.19 [11.53] | 35.60 [7.02] | 36.20 [13.16] |  | 0.28^2^ |
| **SARA sum score**  median [IQR] | 11.00 [8.00, 17.00] | 10.25 [6.88, 21.25] | 15.00 [11.38, 25.88] | 20.00 [15.62, 22.25] | * | 0.01^3^ |
| **SARA sum score**  **annual progression**  median [IQR] | 1.00 [0.00, 2.16] | 1.19 [-0.43, 2.57] | 3.35 [2.07, 4.30] | 0.66 [0.28, 1.67] |  | 0.06^3^ |
| **INAS count**  median [IQR] | 5.00 [3.00, 7.00] | 5.00 [4.00, 8.00] | 4.00 [4.00, 6.00] | 8.00 [5.50, 9.00] |  | 0.20^3^ |
| **Disease duration at baseline, in years^4^**  median [IQR] | 10.81 [5.88 , 16.21] | 9.79 [6.35 , 15.10] | 14.19 [8.36 , 19.44] | 17.50 [12.10 , 21.99] |  | 0.12^3^ |
| ^1^Fisher-Test; ^2^one-way Anova; ^3^Kruskal-Wallis-Test; ^4^disease duration is defined as the time from the age of onset (detailed description is given in the Methods section) until baseline visit, in years; n.a. = non applicable; sign. = significance; IQR = interquartile range; SD = standard deviation; * p < 0.05 | | | | | | |

*Supplementary Table 4.* **Distribution of each INAS item of the ataxic SCA3 mutation carrier***,* **divided per haplotype of the expanded allele**

|  | **Haplotype bp987 – bp1118, expanded allele ataxic SCA3 [N absent / present (%)]** | | | | **sign.** | **p-value^1^** |
| --- | --- | --- | --- | --- | --- | --- |
| **INAS item** | Cytosine - Adenine | Guanine - Cytosine | Cytosine - Cytosine | Guanine - Adenine |  |  |
| **Hyperreflexia** | 94 / 53 (63.9 / 36.1) | 26 / 25 (51.0 / 49.0) | 5 / 6 (45.5 / 54.5) | 7 / 3 (70.0 / 30.0) |  | 0.26 |
| **Areflexia** | 58 / 89 (39.5 / 60.5) | 28 / 23 (54.9 / 45.1) | 9 / 2 (81.8 / 18.2) | 5 / 5 (50.0 / 50.0) | * | 0.02 |
| **Extensor plantar reflex** | 109 / 36 (75.2 / 24.8) | 24 / 24 (50.0 / 50.0) | 5 / 6 (45.5 / 54.5) | 7 / 3 (70.0 / 30.0) | * | 0.01 |
| **Spasticity** | 98 / 46 (68.1 / 31.9) | 23 / 27 (46.0 / 54.0) | 4 / 7 (36.4 / 63.6) | 2 / 5 (28.6 / 71.4) | * | 0.01 |
| **Paresis** | 103 / 43 (70.5 / 29.5) | 31 / 20 (60.8 / 39.2) | 7 / 5 (58.3 / 41.7) | 4 / 6 (40.0 / 60.0) |  | 0.15 |
| **Muscle Atrophy** | 114 / 33 (77.6 / 22.4) | 36 / 15 (70.6 / 29.4) | 11 / 1 (91.7 / 8.3) | 4 / 6 (40.0 / 60.0) | * | 0.03 |
| **Fasciculation** | 123 / 24 (83.7 / 16.3) | 40 / 10 (80.0 / 20.0) | 9 / 3 (75.0 / 25.0) | 6 / 4 (60.0 / 40.0) |  | 0.23 |
| **Myoclonus** | 128 / 19 (87.1 / 12.9) | 36 / 14 (72.0 / 28.0) | 7 / 5 (58.3 / 41.7) | 6 / 4 (60.0 / 40.0) | * | 0.01 |
| **Rigidity** | 139 / 8 (94.6 / 5.4) | 47 / 3 (94.0 / 6.0) | 12 / 0 (100.0 / 0.0) | 9 / 1 (90.0 / 10.0) |  | 0.70 |
| **Chorea and Dyskinesia** | 139 / 8 (94.6 / 5.4) | 48 / 3 (94.1 / 5.9) | 11 / 1 (91.7 / 8.3) | 9 / 1 (90.0 / 10.0) |  | 0.61 |
| **Dystonia** | 119 / 28 (81.0 / 19.0) | 27 / 23 (54.0 / 46.0) | 11 / 1 (91.7 / 8.3) | 3 / 7 (30.0 / 70.0) | ** | < 0.001 |
| **Resting Tremor** | 143 / 4 (97.3 / 2.7) | 51 / 0 (100.0 / 0.0) | 12 / 0 (100.0 / 0.0) | 9 / 1 (90.0 / 10.0) |  | 0.23 |
| **Sensory symptoms** | 34 / 109 (23.8 / 76.2) | 18 / 32 (36.0 / 64.0) | 4 / 8 (33.3 / 66.7) | 3 / 7 (30.0 / 70.0) |  | 0.34 |
| **Urinary dysfunction** | 69 / 78 (46.9 / 53.1) | 27 / 24 (52.9 / 47.1) | 8 / 4 (66.7 / 33.3) | 2 / 8 (20.0 / 80.0) |  | 0.15 |
| **Cognitive impairment** | 111 / 36 (75.5 / 24.5) | 40 / 12 (76.9 / 23.1) | 6 / 6 (50.0 / 50.0) | 6 / 3 (66.7 / 33.3) |  | 0.25 |
| **Brainstem oculomotor signs** | 31 / 118 (20.8 / 79.2) | 17 / 36 (32.1 / 67.9) | 7 / 5 (58.3 / 41.7) | 0 / 9 (0.0 / 100.0) | * | 0.01 |
| ^1^Fisher-Test; sign. = significance; * p < 0.05; ** p < 0.001 | | | | | | |

*Supplementary Table 5.* **Exact Fisher test of the INAS items which showed a significant relation in supplementary table 4, tested for each haplotype of the expanded allele**

|  |  |  |  |
| --- | --- | --- | --- |
| **Haplotype bp987 – bp1118, expanded allele** | **Item** | **sign.** | **p-value^1^** |
| Cytosine - Adenine versus  Guanine - Cytosine | Areflexia |  | 0.07 |
|  | Extensor plantar reflex | * | 0.00 |
|  | Spasticity | * | 0.01 |
| (N= 150)    (N= 54) | Muscle Atrophy |  | 0.35 |
|  | Myoclonus |  | 0.03 |
|  | Dystonia | * | 0.00 |
|  | Brainstem oculomotor signs |  | 0.13 |

|  |  |  |  |
| --- | --- | --- | --- |
| **Haplotype, expanded allele** | **Item** | **sign.** | **p^1^** |
| Cytosine - Adenine  *versus*  Cytosine - Cytosine | Areflexia | * | 0.01 |
|  | Extensor plantar reflex |  | 0.07 |
|  | Spasticity |  | 0.05 |
| (N= 150)    (N= 12) | Muscle Atrophy |  | 0.46 |
|  | Myoclonus |  | 0.02 |
|  | Dystonia |  | 0.70 |
|  | Brainstem oculomotor signs | * | 0.01 |
|  |  |  |  |
| **Haplotype, expanded allele** | **Item** | **sign.** | **p^1^** |
| Cytosine - Adenine  *versus*  Guanine - Adenine | Areflexia |  | 0.52 |
|  | Extensor plantar reflex |  | 0.71 |
|  | Spasticity |  | 0.04 |
| (N= 150)  (N= 10) | Muscle Atrophy | * | 0.02 |
|  | Myoclonus |  | 0.04 |
|  | Dystonia | * | 0.00 |
|  | Brainstem oculomotor signs |  | 0.21 |
|  |  |  |  |
| **Haplotype, expanded allele** | **Item** | **sign.** | **p^1^** |
| Cytosine - Cytosine  *versus*  Guanine - Cytosine | Areflexia |  | 0.17 |
|  | Extensor plantar reflex |  | 1.00 |
|  | Spasticity |  | 0.74 |
| (N= 12)    (N= 54) | Muscle Atrophy |  | 0.27 |
|  | Myoclonus |  | 0.49 |
|  | Dystonia |  | 0.02 |
|  | Brainstem oculomotor signs |  | 0.11 |
|  |  |  |  |
| **Haplotype, expanded allele** | **Item** | **sign.** | **p^1^** |
| Cytosine - Cytosine  *versus*  Guanine - Adenine | Areflexia |  | 0.18 |
|  | Extensor plantar reflex |  | 0.39 |
|  | Spasticity |  | 1.00 |
| (N= 12)    (N= 10) | Muscle Atrophy |  | 0.02 |
|  | Myoclonus |  | 1.00 |
|  | Dystonia | * | 0.01 |
|  | Brainstem oculomotor signs | * | 0.01 |

|  |  |  |  |
| --- | --- | --- | --- |
| **Haplotype, expanded allele** | **Item** | **sign.** | **p^1^** |
| Guanine - Adenine  *versus*  Guanine - Cytosine | Areflexia |  | 1.00 |
|  | Extensor plantar reflex |  | 0.31 |
|  | Spasticity |  | 0.45 |
| (N= 10)  (N= 54) | Muscle Atrophy |  | 0.08 |
|  | Myoclonus |  | 0.47 |
|  | Dystonia |  | 0.30 |
|  | Brainstem oculomotor signs |  | 0.05 |
| ^1^Significancy shown as * p < 0.0167 with corrected ^1^alpha = 0.0167 for multiple comparisons; sign. = significance | | | |
|  | | | |


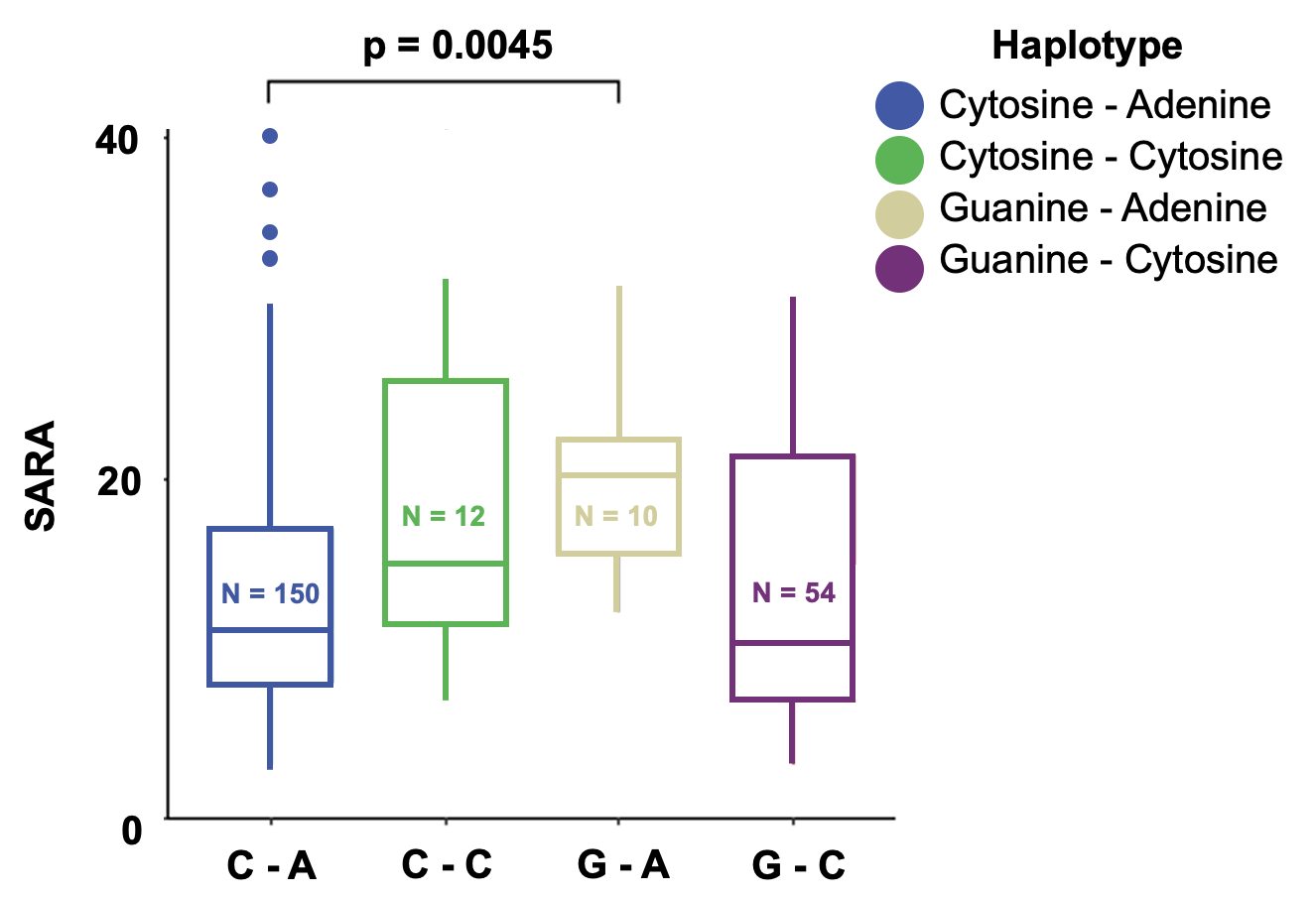


*Supplementary Figure 1.* **Boxplots of ataxia severity assessed with the SARA sum score for each haplotype of the expanded allele at bp987 and bp1118 in ataxic SCA3 mutation carriers.** Kruskal-Wallis-Test showed a statistically significant difference between the haplotypes Cytosine – Adenine (C – A) and Guanine – Adenine (G – A) (p < 0.01). The blue boxplot shows the distribution of Cytosine – Adenine (C – A) (N = 150), the green boxplot the distribution in Cytosine – Cytosine (C – C) (N = 12), the ochre boxplot the distribution in Guanine - Adenine (N = 10) and the purple boxplot the distribution in Guanine – Cytosine (G – C) (N = 54).

*Supplementary Table 6.* **Number of participants with the different haplotypes of the expanded allele given for each research centers ranked by alphabetical order**

|  | **N Haplotype bp987 - bp1118, expanded allele SCA3** | | | | **N total** |
| --- | --- | --- | --- | --- | --- |
| **Research center (in alphabetical order)** | Cytosine - Adenine | Guanine - Cytosine | Cytosine - Cytosine | Guanine - Adenine |  |
| **Aachen** | 2 |  |  |  | 2 |
| **Azores** | 31 | 25 | 8 | 1 | 65 |
| **Bonn** | 37 | 2 | 1 |  | 40 |
| **Coimbra** | 18 | 31 | 2 | 1 | 52 |
| **Essen** | 6 |  | 1 | 1 | 8 |
| **Groningen** | 13 |  |  |  | 13 |
| **Heidelberg** | 7 |  |  |  | 7 |
| **London** | 30 | 8 |  | 6 | 44 |
| **Nijmegen** | 11 |  | 2 | 1 | 14 |
| **Santander** | 7 |  |  |  | 7 |
| **Tübingen** | 27 |  | 1 |  | 28 |
